# Lipid peroxidation of the microsomal fraction and extracted microsomal lipids from DAB-induced hepatomas.

**DOI:** 10.1038/bjc.1979.131

**Published:** 1979-06

**Authors:** T. J. Player, D. J. Mills, A. A. Horton

## Abstract

NADPH- and ascorbic acid-induced microsomal lipid peroxidation was almost absent in subcutaneously implanted DAB-induced hepatomas D23, D30 and D192A, and present at greatly reduced levels in DAB-induced primary hepatomas when compared with normal liver controls. Fatty acid analysis of the microsomal lipid from passaged tumours demonstrated adequate levels of substrate in the phospholipid fractions to support lipid peroxidation. Lipid extracted from hepatoma microsomal fractions was shown to undergo ascorbic acid-induced lipid peroxidation, but to a lesser extent that the corresponding liver extract. This may be partially explained by a decrease in the phospholipid content of hepatoma microsomal membranes. However, phospholipid extracted from microsomal fractions of hepatoma and liver supported lipid peroxidation to a similar extent. The possible role of the non-lipid component of the membrane in the process of lipid peroxidation is discussed.


*Br. J. Cancer* (1979) **39**, 773

### LIPID PEROXIDATION OF THE MICROSOMAL FRACTION AND EXTRACTED MICROSOMAL LIPIDS FROM DAB-INDUCED HEPATOMAS

T. J. PLAYER, D. J. MILLS AND A. A. HORTON

*From the Department of Biochemistry, University of Birmingham, P.O. Box 363, Birmingham B15 2TT*

Received 18 December 1978 Accepted 19 February 1979

**Summary.—NADPH- and ascorbic acid-induced microsomal lipid peroxidation was almost absent in subcutaneously implanted DAB-induced hepatomas D23, D30 and D192A, and present at greatly reduced levels in DAB-induced primary hepatomas when compared with normal liver controls. Fatty acid analysis of the microsomal lipid from passaged tumours demonstrated adequate levels of substrate in the phospholipid fractions to support lipid peroxidation. Lipid extracted from hepatoma microsomal fractions was shown to undergo ascorbic acid-induced lipid peroxidation, but to a lesser extent than the corresponding liver extract. This may be partially explained by a decrease in the phospholipid content of hepatoma microsomal membranes. However, phospholipid extracted from microsomal fractions of hepatoma and liver supported lipid peroxidation to a similar extent. The possible role of the non-lipid component of the membrane in the process of lipid peroxidation is discussed.**

THE DESTRUCTIVE ROLE of lipid peroxides and their decomposition products, and the protection against such damage to the cell afforded by antioxidants is well documented. Examples include studies on the inhibition of mitosis by linoleic acid hydroperoxide (Glushchenko *et al.*, 1975) and the interaction of malonaldehyde (MDA), a breakdown product of lipid peroxidation, with DNA and its inhibitory effect on cell division (Brooks & Klamerth, 1968). Addition of antioxidants to the diet of mice increased their mean life span (Harman, 1968) and the supply of α-tocopherol to fibroblasts *in vitro* produced a 100% increase in the number of population doublings, concomitant with a decrease in the products of lipid peroxidation (Packer & Smith, 1974).

A metabolic role for lipid peroxidation in the cell is unknown. It has been suggested that NADPH-dependent oxidation may contribute to the normal catabolism of phospholipids in microsomal membranes (Poyer & McCay, 1971) and that the peroxidation of polyunsaturated fatty acids mediated by flavin enzymes may contribute to the turnover of membrane lipid components (Fong *et al.*, 1973). Wolfson *et al.* (1956) observed a decrease in lipid peroxidation in regenerating rat liver and proposed an involvement of peroxidation in the regulation of cell division. It has also been suggested that lipid peroxidation may be involved in some types of tumour formation. Shamberger (1972) has shown concomitant lipid peroxidation and skin-tumour formation by topical application of 7,12-dimethylbenzanthracene and croton oil. Prior treatment with antioxidants reduced tumour formation. The effects of antioxidants in reducing the incidence of a variety of chemically induced neoplasias are described by McCay & Poyer (1976).

The extent of lipid peroxidation in cells showing a rapid rate of growth and division is being investigated in this laboratory. It has been shown previously that NADPH-dependent microsomal lipid peroxidation is almost absent in foetal and neonatal rat liver (Player *et al.*, 1977; Slater, 1968). This paper describes experiments designed to investigate both NADPH- and ascorbic acid-induced microsomal lipid peroxidation in some rapidly growing hepatomas.

### MATERIALS AND METHODS

*Animals and hepatomas.*—Hepatomas D23, D30 and D192A were originally a gift from the Cancer Research Campaign Laboratories, University of Nottingham. They were maintained by subcutaneous passage in Wistar rats at the Department of Surgical Immunology, University of Birmingham. Primary hepatoma tissue, induced by feeding 4-dimethylaminoazobenzene (DAB) (Baldwin & Barker, 1967) was obtained directly from the University of Nottingham.

*Chemicals.* — EGTA, Hepes [2-(N-2-hydroxyethylpiperazin-N′-yl) ethanesulphonic acid], Tris, ADP, NADPH, sodium ascorbate, thiobarbituric acid, silicic acid, cytochrome c and bovine serum albumin were supplied by Sigma Chemical Co., Poole, Dorset. Ten per cent EGSS-X on Unisorb AW was from Jones Chromatography Ltd, Llanbradach, Glam., and boron trifluoride-methanol complex was supplied by BDH Chemicals Ltd, Poole, Dorset. All other chemicals used were of the highest purity available commercially.

*Preparation of microsomal fractions.* — Animals bearing s.c. hepatomas were killed by cervical fracture 2–3 weeks after implantation. Tumours were removed and freed from necrotic tissue. Primary hepatomas were freed as much as possible from the surrounding liver tissue, and liver lobes from the same animals that appeared not to contain tumour tissue were also examined. Hepatoma and liver microsomal fractions were prepared by homogenizing the tissues in 0·25M sucrose, 0·5mM EGTA and 5mM Hepes (pH 7·4). The homogenates were centrifuged at ~15,000 $g \times 10$ min in an MSE High Speed 18 centrifuge. The supernatants were removed by Pasteur pipette and centrifuged at ~78,000 $g \times 60$ min in an MSE Super Speed 50 centrifuge. The sedimented fractions were designated the microsomal fractions and were washed in 0·15M Tris-HCl (pH 7·4) and recentrifuged at ~78,000 $g \times 60$ min. The microsomal fractions were finally resuspended in 0·15M Tris-HCl (pH 7·4) unless used for lipid extraction, when they were stored at −20°C under $N_2$ before use.

*Methods for lipid peroxidation.* — For NADPH-dependent microsomal lipid peroxidation the medium consisted of 0·15M Tris-HCl (pH 7·4), 4mM ADP, 1mM $KH_2PO_4$, 0·4mM NADPH, 0·05mM $FeCl_3$ and 1 mg microsomal protein/ml in a total volume of 3 or 4 ml. Samples were incubated at 25°C in a shaking water bath and aliquots (0·5 ml) were taken periodically up to 45 min for assay of malonaldehyde (MDA) formation. Ascorbic acid-induced lipid peroxidation was performed in a medium containing 0·15M Tris-HCl (pH 7·4), 1mM $KH_2PO_4$, 0·25mM ascorbic acid, 0·05mM $FeCl_3$ and 1 mg microsomal protein/ml. Incubation was at 37°C in a shaking water bath, with samples (0·5 ml) being withdrawn at intervals up to 60 min for the assay of MDA. For ascorbic acid-induced peroxidation of extracted microsomal lipids, the lipid (3–6 mg) was taken up in methanol (0·2 ml) and incubated with the ascorbic acid medium (1·0 ml) described above, at 37°C. MDA production in the total incubation volume was measured after 60 min.

*Assay of MDA.*—MDA production was measured by a modification of the thiobarbituric acid test of Hunter *et al.* (1963). Samples taken from the incubation vessels were mixed with distilled water (1·5 ml), 40% trichloracetic acid (0·5 ml), 5N HCl (0·25 ml) and 2% thiobarbituric acid (0·5 ml) and then boiled for 10 min, cooled and centrifuged in an MSE bench centrifuge. The optical densities of the supernatant fractions were recorded at 532 nm. For calculation of specific activity,

$$\epsilon^{1\,\text{cm}}_{532\,\text{nm}} = 1{\cdot}56 \times 10^5 \text{ l/mol/cm}$$

was used as the molar extinction coefficient.

*Lipid analysis.*—Microsomal lipids were extracted according to the method of Folch *et al.* (1957). Separation of the lipid extracts into neutral and phospholipid fractions was carried out using silicic acid columns (1·2 g). Neutral lipids were eluted with chloroform (20 ml) and phospholipids eluted with methanol (25 ml). Methyl ester derivatives of the fatty acids in the fractions were prepared according to Metcalfe & Schmitz (1961) using 14% boron trifluoride-methanol. Fatty acid analysis was performed on a Pye "Series 104" Model 24 gas chromatograph with a flame ionization detector. The column used was

10% EGSS-X on Unisorb AW, which was maintained at 180°C during methyl ester separations.

*Enzyme assay.*—NADPH-cytochrome-c reductase activity was measured by the procedure of Phillips & Langdon (1962) using

$$\epsilon^{1\,cm}_{550\,nm} = 18{\cdot}5 \times 10^3\ l/mol/cm$$

as the molar extinction coefficient. Microsomal fractions used for this assay were prepared in 0·1M sodium phosphate buffer (pH 7·6). Protein estimation was by the method of Lowry *et al.* using bovine serum albumin as standard.

### RESULTS

Microsomal lipid peroxidation was shown to be either absent or present at extremely low levels in all the s.c. hepatomas examined (Table I). Production of MDA was low in these fractions, whether the source of electrons for the reaction was NADPH or ascorbic acid. MDA levels increased slightly when the microsomal fraction from primary hepatomas was subjected to peroxidizing conditions. This may have resulted from contamination with liver tissue, as primary tumours were only freed from contaminating liver cells by visual inspection. The microsomal fraction isolated from the liver tissue of primary hepatoma-bearing animals showed a slightly greater production of MDA than the controls. This may be due to variation in diet.

TABLE I.—*Microsomal lipid peroxidation induced by the NADPH system or the ascorbic-acid system**

| | MDA production (nmol/mg protein)† | |
|---|---|---|
| Tissue | NADPH | Ascorbic acid |
| Liver | 33·0 | 35·8 |
| D23 | 0·21 | 0·42 |
| D30 | 0·58 | 1·04 |
| D192A | 0·00 | 0·42 |
| D30 (heat-treated)‡ | — | 1·04 |
| Liver from DAB-treated rats | 38·7 | 40·8 |
| Primary DAB-induced hepatoma | 3·3 | 5·4 |

* Means of 2 separate experiments each using a microsomal fraction from a different animal and performed in duplicate.
† After incubation for 45 min.
‡ Microsomal fraction was heated at 80°C for 5 min in a water bath.

NADPH-cytochrome-c reductase is known to be involved as an electron carrier in liver microsomal NADPH-dependent lipid peroxidation (Pederson *et al.*, 1973). The activity of this enzyme in microsomal fractions from hepatomas was much less than in corresponding liver fractions (Table II).

TABLE II.—*Activity of NADPH-cytochrome-c reductase in microsomal fractions from liver and hepatomas**

| Tissue | NADPH-cytochrome-c reductase activity (nmol/mg protein/min) |
|---|---|
| Liver | 122·3 ± 10·9 |
| D23 | 16·9 ± 5·8 |
| D30 | 14·1 ± 6·1 |
| D192A | 9·0 ± 4·3 |

* Values are expressed as means ± s.d. for 3 separate experiments each using one animal.

The principal substrate for lipid peroxidation, arachidonic acid (Niehaus & Samuelson, 1968) was found almost entirely in the phospholipid fraction of all the microsomal lipid fractions examined (Table III). The proportion of phospholipid to neutral lipid was found to be lower in microsomal lipid from all hepatomas

TABLE III.—*Analysis of microsomal lipid from liver and hepatomas**

| | Fatty acid composition | | | | | PL content (%) |
|---|---|---|---|---|---|---|
| | 16:0 | 18:0 | 18:1 | 18:2 | 20:4 | |
| Liver | | | | | | |
| NL† | 36·1‡ | 27·1 | 14·7 | 22·1 | —§ | 90 |
| PL | 29·2 | 26·7 | 16·2 | 10·2 | 17·7 | |
| D23 | | | | | | |
| NL | 26·6 | 36·4 | 27·4 | 9·6 | — | 79 |
| PL | 24·5 | 27·2 | 25·1 | 8·9 | 14·3 | |
| D30 | | | | | | |
| NL | 30·3 | 35·7 | 19·5 | 12·2 | 2·3 | 75 |
| PL | 22·9 | 32·9 | 19·0 | 6·9 | 18·3 | |
| D192A | | | | | | |
| NL | 31·6 | 26·8 | 24·1 | 17·5 | — | 60 |
| PL | 22·5 | 22·3 | 20·1 | 10·4 | 24·7 | |

* Averages of 2 experiments each using one animal and performed in duplicate.
† NL = neutral lipid, PL = phospholipid.
‡ Peak area percentages.
§ < 1% of total fatty acids.

than from liver, the greatest decrease being shown by hepatoma D192A, where phospholipids accounted for 60% of the total, compared with 90% in liver.

Extracted lipid from hepatoma microsomal fractions as well as that from liver microsomal preparations was shown to support ascorbic acid-induced peroxidation (Table IVa). MDA production/mg of hepatoma microsomal lipid was less than 40% of the mean value when liver microsomal lipids were used as substrate. Separation of the extracted lipid into phospholipid and neutral lipid fractions, followed by incubation with the ascorbic acid peroxidation system, demonstrated that the phospholipid fraction was responsible for most of the lipid peroxidation (Table IVb). There is no significant difference between MDA production/mg phospholipid from liver and that from hepatoma samples.

TABLE IV.—*Ascorbic acid-induced malonaldehyde production in extracted microsomal lipids from liver and hepatomas**

(a) Total extracted lipid

| | MDA production ($OD_{532}$) |
|---|---|
| Liver (6) | 0·473±0·120 |
| D30 (4) | 0·181±0·105 |
| D192A (3) | 0·160±0·100 |

(b) Phospholipid and neutral lipid

| | MDA production ($OD_{532}$) | |
|---|---|---|
| | PL | NL |
| Liver (4) | 0·545±0·150 | 0·075±0·025 |
| D30 (4) | 0·420±0·100 | 0·055±0·020 |
| D192A (3) | 0·380±0·126 | 0·065±0·030 |

* Results are expressed as mean±s.d. and the numbers of samples from separate animals are shown in parentheses.

### DISCUSSION

Analysis of the fatty acids of the hepatoma microsomal phospholipid fractions showed some similarity to the fatty acid profile of liver microsomal phospholipids. From these data it would appear that there is adequate arachidonic acid present in hepatoma phospholipid to support microsomal lipid peroxidation, even though the overall percentage of phospholipid in the lipids of the membrane is decreased.

NADPH-dependent lipid peroxidation, which is mediated by NADPH-cytochrome-c reductase, and is active in liver microsomal fractions from both control animals and animals bearing primary DAB-induced tumours, was absent from the s.c. hepatomas examined. It is unlikely that the lower activities of the reductase found in these hepatoma microsomal fractions are entirely responsible for the lack of NADPH-dependent lipid peroxidation, because even in the presence of the non-enzymic ascorbic acid-induced peroxidation system, none of the passaged hepatoma microsomal fractions examined produced measurable amounts of MDA.

Extracted lipid from hepatoma microsomal fractions was shown to be capable of supporting lipid peroxidation. However, it was also evident that the production of MDA was much less than for the corresponding liver fraction. Separation of the lipids into phospholipid and neutral lipid components demonstrated that the phospholipid component was mainly responsible for peroxidation in all fractions examined. This is in agreement with the distribution of arachidonic acid as shown chromatographically. The lower production of MDA with the total lipid extracts from the hepatomas may be partially explained by the decreased percentage of phospholipid found in the membrane. In view of the observation that lipids extracted from hepatoma microsomal fractions supported peroxidation, the absence of MDA production on incubation of hepatoma microsomal fractions with either the enzymic or non-enzymic peroxidation-induction systems indicates some inhibitory factor(s). Heat treatment of the hepatoma microsomal fractions produced no increase in lipid peroxidation in the presence of the ascorbic acid system (Table I), thus eliminating the possibility that the inhibitory factor is a protein. It is also unlikely that inhibition of lipid peroxidation is due to membrane conformation protecting the unsaturated fatty acids, as heat treatment would most probably result in the disruption of such organization.

The role of the non-lipid portion of the membrane may prove to be extremely important in its effect on lipid peroxidation. Sharma (1977) has shown that when the protein fraction of rat-brain mitochondria is added back to extracted lipid, ascorbic acid-induced peroxidation may be stimulated by over 100%. By using the approximation that 100 nmol MDA originates from 890 nmol fatty acid (May & McCay, 1968), it can be calculated that 220 nmol fatty acid/mg lipid was utilized during the course of ascorbic acid-induced peroxidation in the liver microsomal vesicles, whereas only 36 nmol fatty acid/mg lipid was used when extracted lipid was the substrate. Thus, the non-lipid liver microsomal fraction appears to be involved in catalysis of the reaction. This is obviously not the case with the hepatoma fractions examined above, and it would appear that an antioxidant activity is incorporated into this fraction. Experiments to investigate the nature of the total inhibition of hepatoma microsomal preparations are in progress.

The results reported here are another example of the absence of microsomal lipid peroxidation in rapidly growing cells. NADPH-dependent microsomal lipid peroxidation has been shown to be decreased by 30% in regenerating liver 48 h after partial hepatectomy (unpublished observation), and to be virtually inactive in the foetal and neonatal fractions (Player *et al.*, 1977). It may be that lipid peroxide production in the cell is in some way connected with the regulation of division and growth.

Experiments of short duration in which dimethylnitrosamine was added to a suspension of rat-liver microsomes produced an increase in NADPH-dependent lipid peroxidation by stimulation of mixed-function oxidase (Jose & Slater, 1970). In contrast, microsomal lipid peroxidation was found to be extremely low in all the chemically induced hepatomas investigated in this laboratory, which resulted from long-term treatment of rats with carcinogens. Thiele & Huff (1960) also found that the majority of mitochondrial fractions from the tumours investigated had very little ascorbate-induced lipid peroxidation. It was observed in this laboratory that very little MDA was produced when mitochondrial fractions and whole homogenates of DAB-induced hepatomas were incubated with the ascorbic-acid peroxidation system (unpublished observations). In conclusion, some chemical carcinogens may show a correlation between initiation of lipid peroxidation and neoplasia, but in the case of the passaged, rapidly growing, chemically induced hepatomas examined in this laboratory, there is a complete absence of peroxidation potential. Thus although lipid peroxidation and free radicals may damage cells by reaction with DNA, and this may also be the mode of action at the time of neoplastic induction, peroxidation systems do not persist in the tumour itself, and would appear to be unnecessary for cell proliferation.

This work was supported by a grant from the Cancer Research Campaign.

### REFERENCES

BALDWIN, R. W. & BARKER, C. R. (1967) Tumour-specific antigenicity aminoazo-dye-induced rat hepatomas. *Int. J. Cancer*, **2**, 355.

BROOKS, B. R. & KLAMERTH, O. C. (1968) Interaction of DNA with bifunctional aldehydes. *Eur. J. Biochem.*, **5**, 178.

FOLCH, J., LEES, M. & SLOANE-STANLEY, G. H. (1957) A simple method for the isolation and purification of total lipids from animal tissues. *J. Biol. Chem.*, **226**, 497.

FONG, K., MCCAY, P. B. & POYER, J. L. (1973) Evidence that peroxidation of lysosomal membranes is initiated by hydroxyl free radicals produced during flavin enzyme activity. *J. Biol. Chem.*, **248**, 7792.

GLUSHCHENKO, N. N., SHESTAKOVA, S. V. & DANILOV, V. S. (1975) Effects of lipid peroxides on cell division. *Biol. Nauki*, **18**, 51.

HARMAN, D. (1968) Free radical theory of ageing: Effect of free radical reaction inhibitors on the mortality rate of male $LAF_1$ mice. *J. Gerontol.*, **23**, 476.

HUNTER, F. E., GEBICKI, J. M., HOFFSTEN, P. E., WEINSTEIN, J. & SCOTT, A. (1963) Swelling and lysis of rat liver mitochondria induced by ferrous ions. *J. Biol. Chem.*, **238**, 828.

JOSE, P. J. & SLATER, T. F. (1970) The effects of various inhibitors on the stimulation of lipid peroxidation produced by dimethylnitrosamine in rat liver microsomes *in vitro*. *Biochem. J.*, **117**, 66P.

May, H. E. & McCay, P. B. (1968) Reduced triphosphopyridine nucleotide oxidase-catalysed alterations of membrane phospholipids. II. Enzymic properties and stoichiometry. *J. Biol. Chem.*, **243**, 2296.

McCay, P. B. & Poyer, J. L. (1976) Enzyme-generated free radicals as initiators of lipid peroxidation in biological membranes. In *The Enzymes of Biological Membranes*, Vol. 4, Ed. A. Martonosi. London: J. Wiley & Sons. p. 239.

Metcalfe, L. D. & Schmitz, A. A. (1961) The rapid preparation of fatty acid esters for gas chromatographic analysis. *Anal. Chem.*, **33**, 363.

Niehaus, W. G. & Samuelson, B. (1968) Formation of malonaldehyde from phospholipid arachidonate during microsomal lipid peroxidation. *Eur. J. Biochem.*, **6**, 126.

Packer, L. & Smith, J. R. (1974) Extension of the lifespan of cultured normal human diploid cells by vitamin E. *Proc. Natl Acad. Sci. USA*, **71**, 4763.

Pederson, T. C., Buege, J. A. & Aust, S. D. (1973) Microsomal electron transport. The role of reduced nicotinamide adenine dinucleotide phosphate-cytochrome c reductase in liver microsomal lipid peroxidation. *J. Biol. Chem.*, **248**, 7134.

Phillips, A. H. & Langdon, R. G. (1962) Hepatic triphosphopyridine nucleotide-cytochrome c reductase: Isolation, characterization, and kinetic studies. *J. Biol. Chem.*, **237**, 2652.

Player, T. J., Mills, D. J. & Horton, A. A. (1977) Age-dependent changes in rat liver microsomal and mitochondrial NADPH-dependent lipid peroxidation. *Biochem. Biophys. Res. Commun.*, **78**, 1397.

Poyer, J. L. & McCay, P. B. (1971) Reduced triphosphopyridine nucleotide oxidase-catalysed alterations of membrane phospholipids. IV. Dependence on $Fe^{3+}$. *J. Biol. Chem.*, **246**, 263.

Shamberger, R. J. (1972) Increase of peroxidation in carcinogenesis. *J. Natl Cancer Inst.*, **48**, 1491.

Sharma, O. P. (1977) Peroxidation of rat brain mitochondrial lipids. *J. Neurochem.*, **28**, 1377.

Slater, T. F. (1968) The inhibitory effects *in vitro* of phenothiazine and other drugs on lipid peroxidation systems in rat liver microsomes and their relationship to the liver necrosis produced by carbon tetrachloride. *Biochem. J.*, **106**, 155.

Thiele, E. H. & Huff, J. W. (1960) Lipid peroxide production and inhibition by tumour mitochondria. *Arch. Biochem. Biophys.*, **88**, 208.

Wolfson, N., Wilbur, K. M. & Bernheim, F. (1956) Lipid peroxide formation in regenerating rat liver. *Exp. Cell Res.*, **10**, 556.


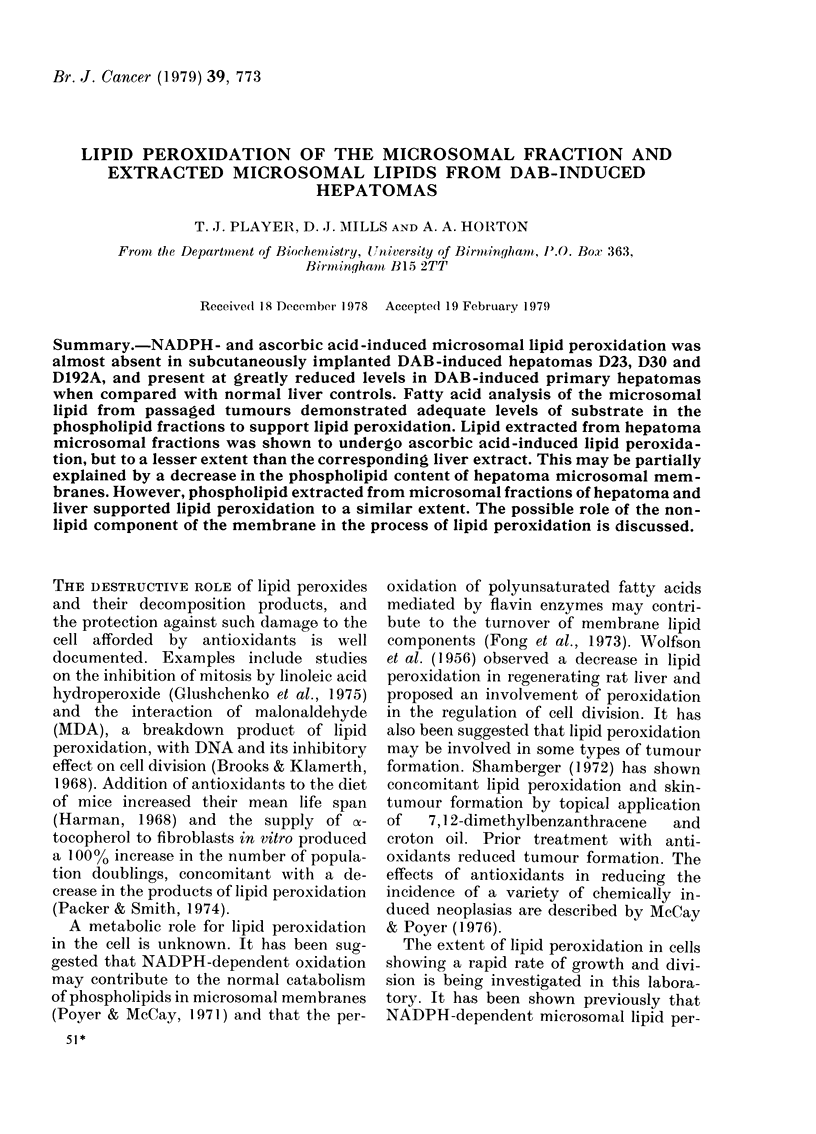

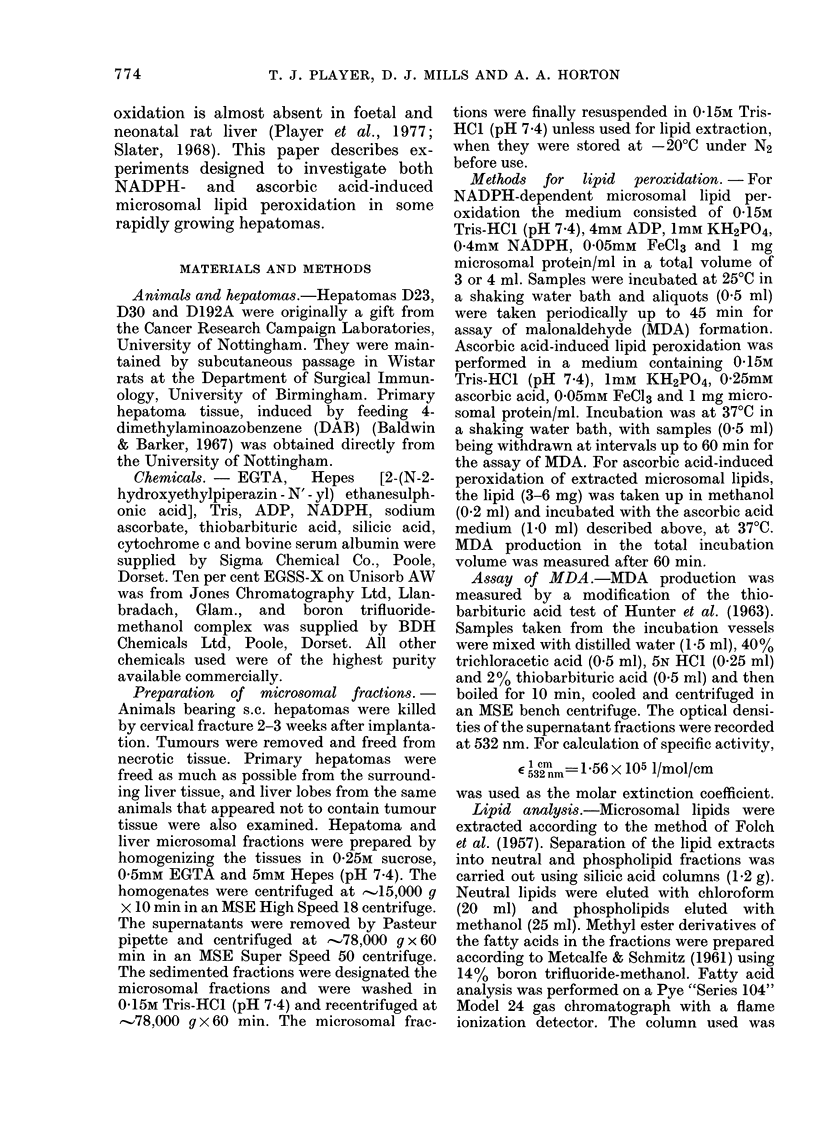

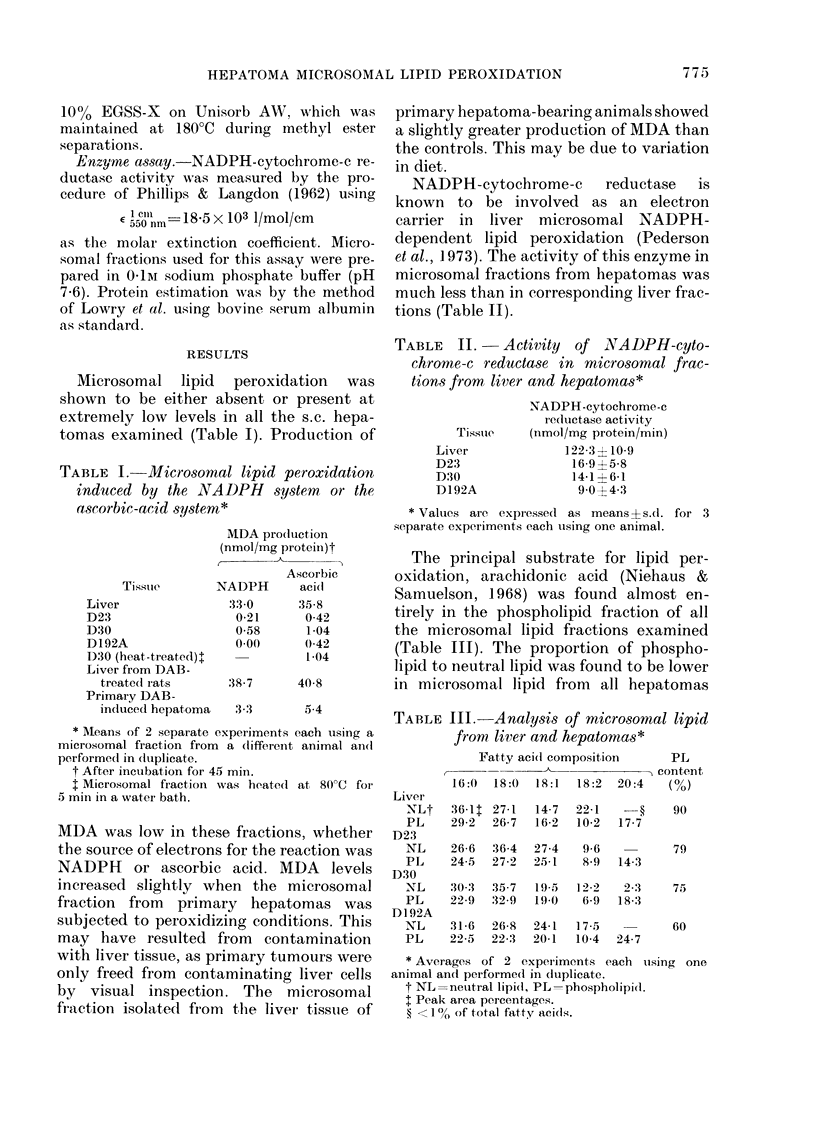

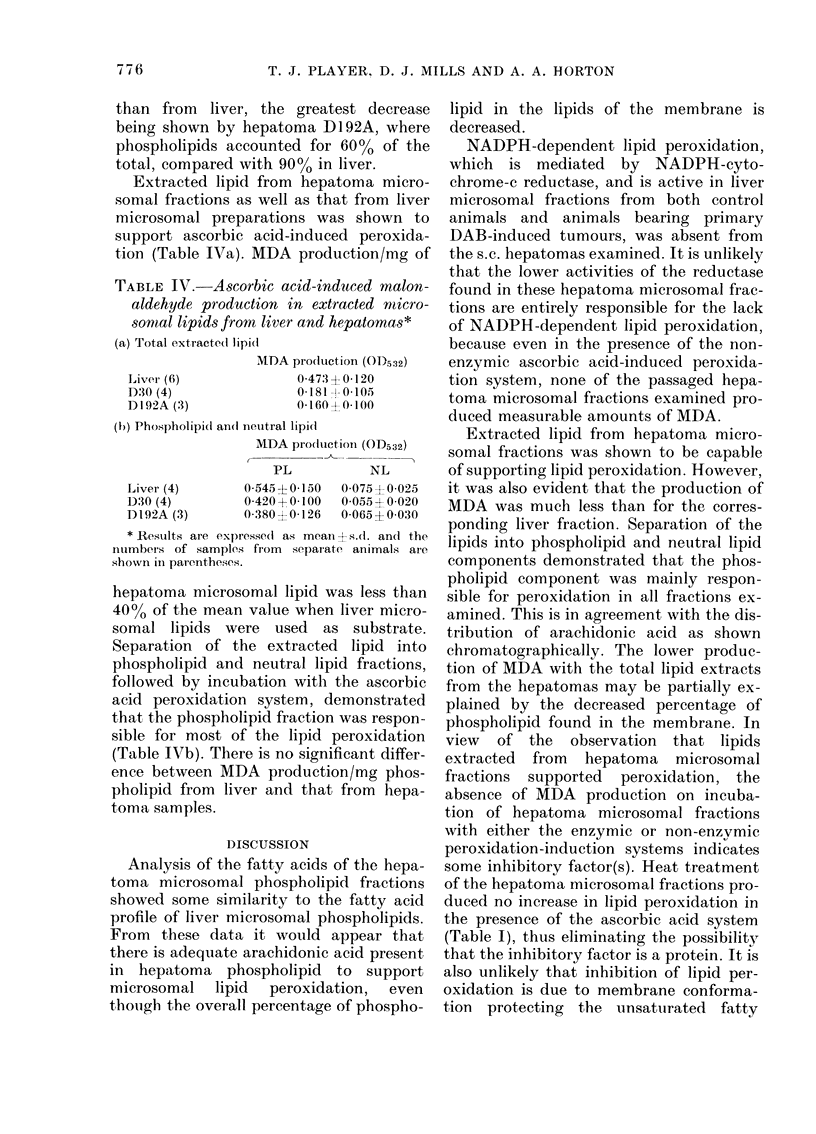

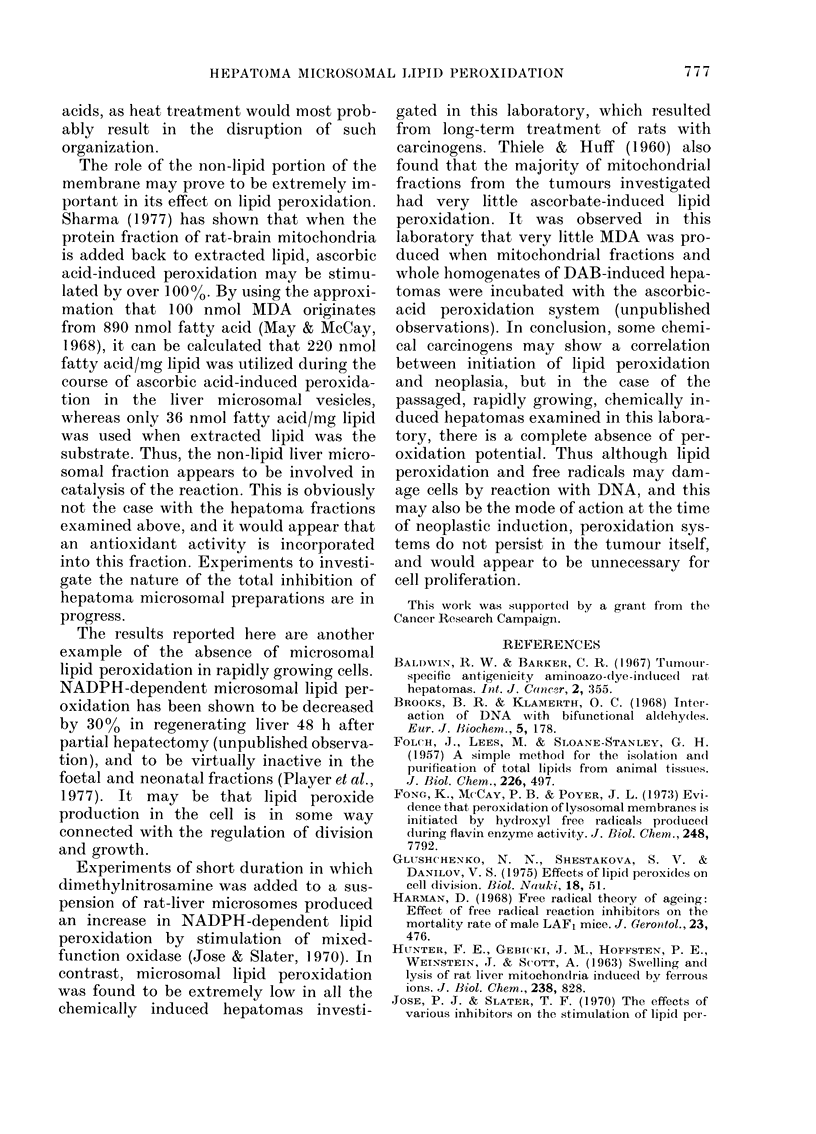

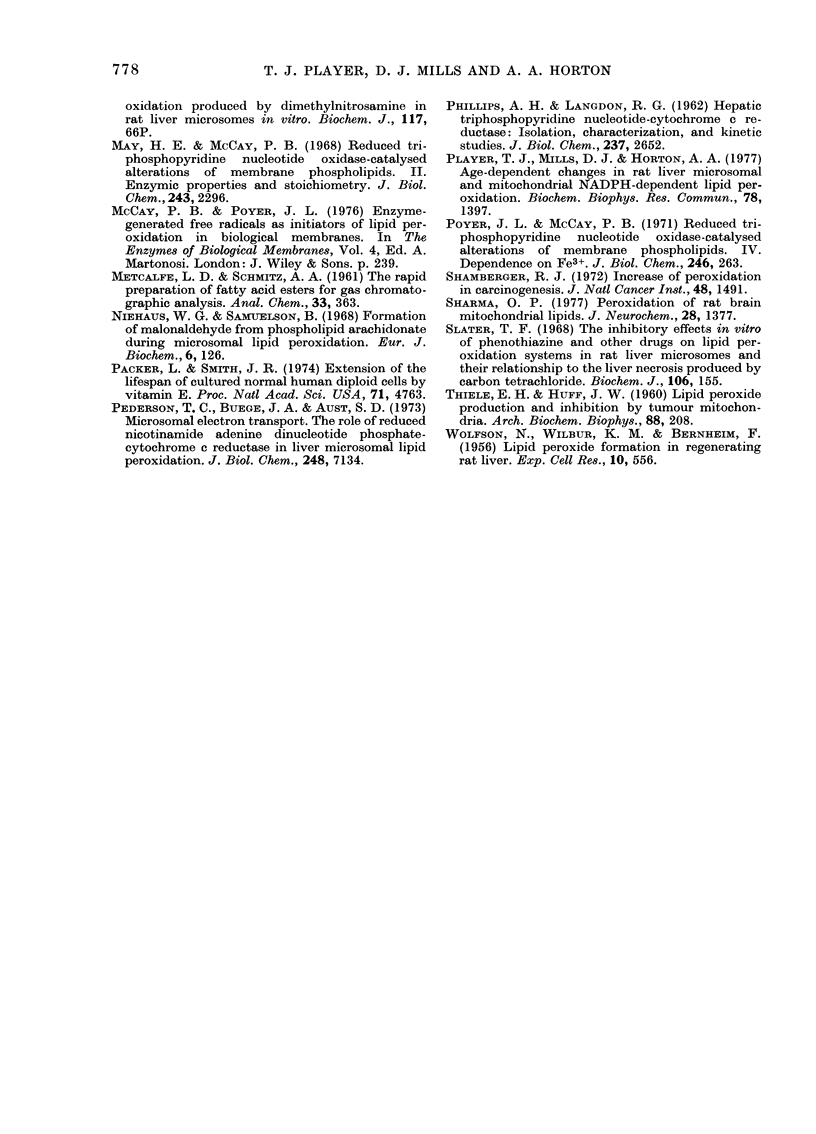


## References

[OCR_00619] Brooks B. R., Klamerth O. L. (1968). Interaction of DNA with bifunctional aldehydes.. Eur J Biochem.

[OCR_00624] FOLCH J., LEES M., SLOANE STANLEY G. H. (1957). A simple method for the isolation and purification of total lipides from animal tissues.. J Biol Chem.

[OCR_00630] Fong K. L., McCay P. B., Poyer J. L., Keele B. B., Misra H. (1973). Evidence that peroxidation of lysosomal membranes is initiated by hydroxyl free radicals produced during flavin enzyme activity.. J Biol Chem.

[OCR_00648] HUNTER F. E., GEBICKI J. M., HOFFSTEN P. E., WEINSTEIN J., SCOTT A. (1963). Swelling and lysis of rat liver mitochondria induced by ferrous ions.. J Biol Chem.

[OCR_00642] Harman D. (1968). Free radical theory of aging: effect of free radical reaction inhibitors on the mortality rate of male LAF mice.. J Gerontol.

[OCR_00654] Jose P. J., Slater T. F. (1970). The effects of various inhibitors on the stimulation of lipid peroxidation produced by dimethylnitrosamine in rat liver microsomes in vitro.. Biochem J.

[OCR_00664] May H. E., McCay P. B. (1968). Reduced triphosphopyridine nucleotide oxidase-catalyzed alterations of membrane phospholipids. II. Enzymic properties and stoichiometry.. J Biol Chem.

[OCR_00683] Niehaus W. G., Samuelsson B. (1968). Formation of malonaldehyde from phospholipid arachidonate during microsomal lipid peroxidation.. Eur J Biochem.

[OCR_00700] PHILLIPS A. H., LANGDON R. G. (1962). Hepatic triphosphopyridine nucleotide-cytochrome c reductase: isolation, characterization, and kinetic studies.. J Biol Chem.

[OCR_00689] Packer L., Smith J. R. (1974). Extension of the lifespan of cultured normal human diploid cells by vitamin E.. Proc Natl Acad Sci U S A.

[OCR_00693] Pederson T. C., Buege J. A., Aust S. D. (1973). Microsomal electron transport. The role of reduced nicotinamide adenine dinucleotide phosphate-cytochrome c reductase in liver microsomal lipid peroxidation.. J Biol Chem.

[OCR_00706] Player T. J., Mills D. J., Horton A. A. (1977). Age-dependent changes in rat liver microsomal and mitochondrial NADPH-dependent lipid peroxidation.. Biochem Biophys Res Commun.

[OCR_00713] Poyer J. L., McCay P. B. (1971). Reduced triphosphopyridine nucleotide oxidase-catalyzed alterations of membrane phospholipids. IV. Dependence on Fe3+.. J Biol Chem.

[OCR_00719] Shamberger R. J. (1972). Increase of peroxidation in carcinogenesis.. J Natl Cancer Inst.

[OCR_00723] Sharma O. P. (1977). Peroxidation of rat brain mitochondrial lipids.. J Neurochem.

[OCR_00727] Slater T. F. (1968). The inhibitory effects in vitro of phenothiazines and other drugs on lipid-peroxidation systems in rat liver microsomes, and their relationship to the liver necrosis produced by carbon tetrachloride.. Biochem J.

[OCR_00734] THIELE E. H., HUFF J. W. (1960). Lipide peroxide production and inhibition by tumor mitochondria.. Arch Biochem Biophys.

[OCR_00739] WOLFSON N., WILBUR K. M., BERNHEIM F. (1956). Lipid peroxide formation in regenerating rat liver.. Exp Cell Res.

